# Apatite calcific periarthritis of the radial collateral ligament of the thumb: a case report and review of the literature

**DOI:** 10.1080/23320885.2019.1654871

**Published:** 2019-08-23

**Authors:** Wassim Zribi, Mohamed Mokhtar Jmal, Ameur Abid, Mohamed Ben Jemaa, Nabil Krid, Mohamed Zribi, Hassib Keskes

**Affiliations:** CHU Habib Bourguiba, Sfax, Tunisia

**Keywords:** Apatite, hand, metacarpophalangeal thumb, ultrasound

## Abstract

Calcification of the lateral collateral ligament of the metacarpophalangeal thumb is a rare pathology. It’s may be due to a deposit of hydroxyapatite crystals. We report the case of a 36-year-old man with chronic pain of MCPP. X-ray and Ultrasound finds a calcium deposit of LCL.

## Aim

Acute calcific tendinitis (ACT), also known as peritendinitis or calcified periarthritis, is an acute inflammatory condition of unknown aetiology commonly occurring in the pericapsular region of the shoulder and less commonly in the hand or wrist. Calcification of the lateral collateral ligament (LCL) of the metacarpophalangeal thumb (MCPP) is a very rare pathology. This calcification may be due to a deposit of hydroxyapatite crystals falling within the framework of apatitic rheumatism of the hand. Through an observation of calcific periarthritis with apatite of the hand and the review of the literature, we present the clinical, radiological and therapeutic aspects of this pathology.

## Material

**Figure 1.**
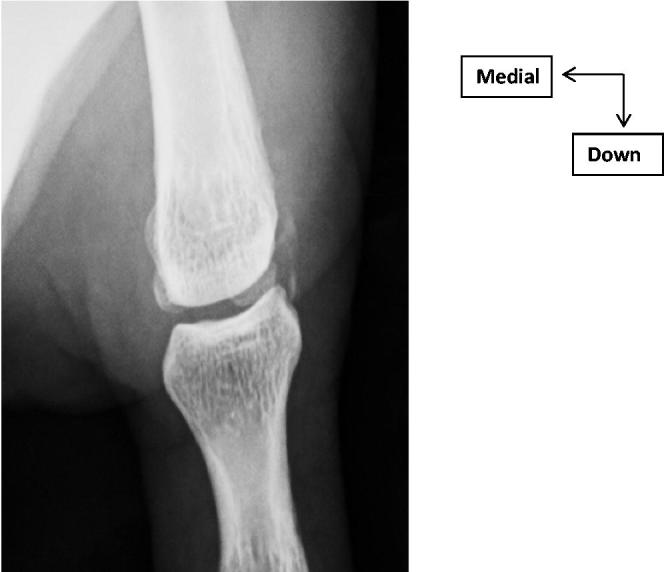
Inches x-ray: calcification of LCL of MCPP.

**Figure 2.**
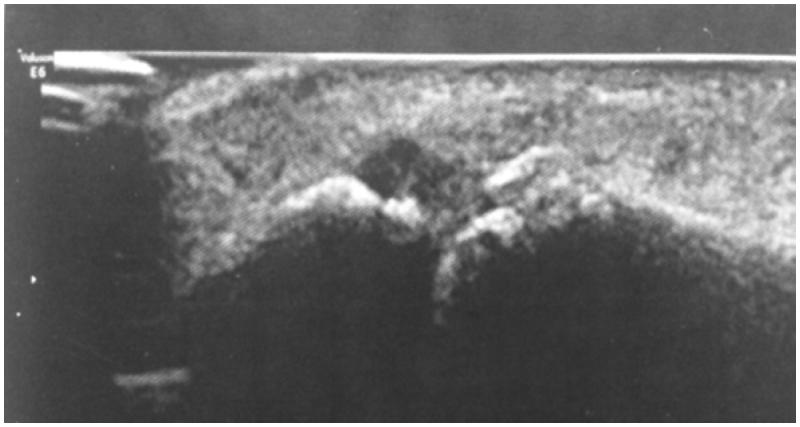
Thumb ultrasound: calcified appearance of MCPL LCL with synovial hypertrophy.

**Figure 3.**
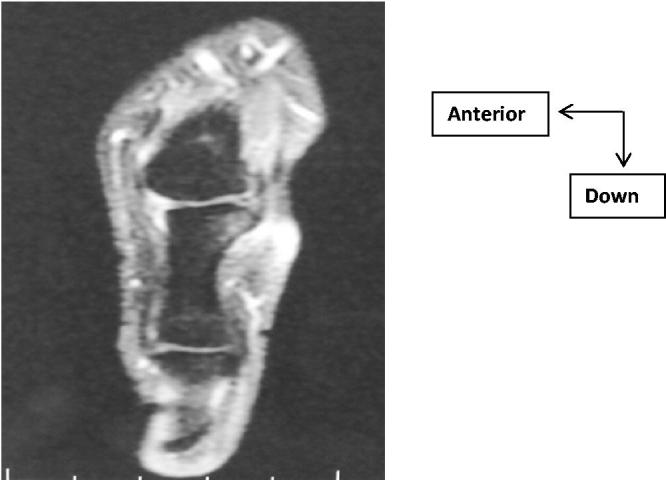
MRI: (sagittal section fat sat T2) Thickened aspect of LCL of MCPP which is in Hypo-signal T1 Hypo-signal T2.

A 36-year-old man with chronic pain of MCPP. The examination found an inflammatory appearance with oedema, redness and pain on palpation of LCL of MCPP. The mild mobilisation of MCPP was painless. The inflammatory and phosphocalcic balance was normal. Standard X-ray showed LPC calcification of MCPP (Figure 1). Ultrasound finds a calcium deposit of LCL (Figure 2). These lesions were better analysed by magnetic resonance imaging of the thumb (Figure 3). In addition, the radiological exploration of the other joints was normal. A symptomatic treatment based on analgesics and anti-inflammatory drugs with icing was introduced. Immobilisation with a thumb orthosis was maintained for 3 weeks. The patient found, after the conservative treatment, a clear clinical and radiological improvement with regression of the thumb pains and a normal function of the hand.

## Discussion

Periarticular calcifications with hydroxyapatite deposits were described in 1966 by Mac Carthy and Gatter [1]. The pathogenesis of this rare disease is uncertain, but two hypotheses exist. There may be local stress as a response to local necrosis resulting from microtrauma, causing calcium deposition and inflammatory reaction [2–4] Uhthoff and all [5], however, have shown no evidence of inflammatory infiltration or scarring. were not seen in a series of 46 cases of calcific tendinitis treated surgically. They suggest that tendon hypoxia is the inciting event, with poor vascular perfusion caused by mechanical or metabolic problems, factors leading to tendon transformation into fibrocartilage where chondrocytes mediate calcium deposition.

Hydroxyapatite calcifications affect men and women equally and are particularly common between 40 and 60 years of age [6].

The disease is usually mono-articular and most often asymptomatic of chance discovery. They can be revealed by acute periarticular inflammation with intense pain preventing mobilisation of the joint and sometimes accompanied by swelling and general signs (fever) with non-specific biological inflammatory syndrome, and this for a few days [7]. Rarely, calcifications are responsible for chronic pain.

Localizations of apatitic calcifications in the hand are rare. They sit near the tendon of the carpi-ulnar flexor muscle, in the vicinity of the pisiform bone.

They also concern the tendon of the carpal radial flexor opposite the palmar face of the radiocarpal spacing. Hydroxyapatite deposits also have a predilection for the metacarpophalangeal and interphalangeal proximal joints of the fingers [8,9].

LCL of MCPP is a rare localisation of apatitic rheumatism.

The diagnosis is established by a hand radiography showing the image of amorphous, homogeneous calcium density cluster. Their contours are smooth without cortical and their forms are often ovoid, sometimes triangular, rarely linear [10]. These characteristics make it possible to differentiate them at the level of the hands, but also from the feet, from possible sesamoïdes, or from bone removals. Ultrasound serves as a convenient, non-invasive imaging tool with superb soft tissue resolution. It can detect tendinous calcifications, even at the beginning of the formation phase [10]. It always remains dependent from the operator.

Computed tomography is necessary, not for the positive diagnosis, but to specify the topography of calcification most often in a zone of tendinous insertion, and for the exploration of cortical erosions.

MRI retains its place in the presence of such calcifications on standard radiographs to diagnose this entity and avoid imaging traps. In addition, it provides a precise description of the morphology and location of calcification and its effect on surrounding structures that may be useful in guiding therapeutic management [10].

The main differential diagnoses to be eliminated are septic arthritis, gouty arthropathies, calcified circumscribed myositis, metabolic abnormalities such as hypervitaminosis D, chronic renal failure, hyperparathyroidism [10].

Symptomatic treatment with nonsteroidal anti-inflammatory drugs and colchicine is sufficient for recovery. After some painful breakouts, these calcifications can disappear spontaneously. Their persistence indicates surgical excision.

An appropriate diagnosis with a correct therapeutic behaviour is the only guarantors of the good results. Any periarticular pain or ligament insertion in the hand should encourage practitioners to request a standard radiological assessment for calcification in favour of an apatitic deposit.
